# Microwave-Assisted Vacuum Synthesis of TiO_2_ Nanocrystalline Powders in One-Pot, One-Step Procedure

**DOI:** 10.3390/nano12010149

**Published:** 2021-12-31

**Authors:** Enrico Paradisi, Roberto Rosa, Giovanni Baldi, Valentina Dami, Andrea Cioni, Giada Lorenzi, Cristina Leonelli

**Affiliations:** 1 Department of Engineering “Enzo Ferrari” (DIEF), University of Modena and Reggio Emilia, 41125 Modena, Italy; cristina.leonelli@unimore.it; 2 Department of Sciences and Methods for Engineering, University of Modena and Reggio Emilia, 42122 Reggio Emilia, Italy; roberto.rosa@unimore.it; 3 Ce.Ri.Col. Colorobbia Research Centre, Colorobbia Consulting S.R.L., 50059 Sovigliana-Vinci, Italy; baldig@colorobbia.it (G.B.); damiv@colorobbia.it (V.D.); cionia@colorobbia.it (A.C.); lorenzig@colorobbia.it (G.L.)

**Keywords:** TiO_2_ nanoparticles, synthesis under vacuum, microwaves, sol–gel synthesis

## Abstract

A new method for fast and simple synthesis of crystalline TiO_2_ nanoparticles with photocatalytic activity was developed by carrying out a classic sol–gel reaction directly under vacuum. The use of microwaves for fast heating of the reaction medium further reduces synthesis times. When the solvent is completely removed by vacuum, the product is obtained in the form of a powder that can be easily redispersed in water to yield a stable nanoparticle suspension, exhibiting a comparable photocatalytic activity with respect to a commercial product. The present methodology can, therefore, be considered a process intensification procedure for the production of nanotitania.

## 1. Introduction

The market demand for TiO_2_ nanopowders is continuously growing [1,2,3], due to the interesting properties of this material. Indeed, nanotitania is widely employed as a photocatalyst for coating applications in many sectors [4,5,6], and intensive academic research has been carried out in order to improve the properties of this material [7]. Nevertheless, simple TiO_2_ photocatalysts are still widely employed because of their robustness and reliability and became standard products in the industry for many applications [8,9,10]. In the need for larger production of this feedstock, new, fast, and efficient processes are always highly desired, in order to increase production, reduce costs and develop greener syntheses, according to process intensification principles [11,12,13]. Microwave (MW) heating technology can be classified as process intensification technology since its faster heating generally results in power and time-saving processes with respect to conventional heating [14,15,16]. For this reason, exploring MW heating and other methods for the fast synthesis of TiO_2_ nanoparticles can be valuable research for the chemical industry. Given our interest in MW-assisted TiO_2_ synthesis, in our previous paper [17], we designed a modified MW system for the synthesis of TiO_2_ nanoparticles, with the possibility of microwave-aided solvent distillation as a post-processing step. In that paper, we proved that the distillation of isopropanol is beneficial both for the obtainment of low nanoparticle size and for suspension stability, as already proved for nano TiO_2_ solvothermal preparation by other researchers [18]. In this paper, we extend our work one step further, with the development of a new TiO_2_ MW-assisted synthesis directly carried out under vacuum. The vacuum has been previously used in TiO_2_ treatments mainly in a second step carried out after its synthesis, in order, for example, to dry the material [19,20,21,22,23], anneal it [24,25], and impregnate selected substrates with this product [26,27,28], together with further post-synthetic treatments [29,30,31,32]. The novelty of this research work lies in the proposed MW plus vacuum combined approach, given that investigation on direct TiO_2_ synthesis under vacuum is sporadic [33], and sometimes not applicable for large-scale production [34], despite a known procedure is available for silica [35]. The application of vacuum distillation during this synthesis is useful to speed up the reaction and favor its completion in short times, since the removal of isopropanol (i-PrOH), one of the reaction products, by distillation will shift the equilibrium on the right, as depicted in Equation (1).

(1) $$\mathrm{Ti}{\left(\mathrm{OiPr}\right)}_{4}+2{\;H}_{2}O\;\overset{\mathrm{HCl}}{\to }{\;\mathrm{TiO}}_{2}+4\;\mathrm{iPrOH}↑$$

This procedure was designed as a technology transfer, with the aim to reduce production times in the preparation of an industrial TiO_2_ product maintaining its well-defined features in terms of nanoparticle size, photocatalytic activity, crystallinity, and suspension’s stability. With this paper, we show that the vacuum sol–gel synthesis of TiO_2_ nanoparticles can be performed in a one-pot, one-step approach, and this can be an effective, fast, and reliable method to obtain crystalline nanoparticles with a good photocatalytic activity that, in principle, can be scaled up for large-scale production.

## 2. Materials and Methods

### 2.1. Experimental Setup

**Figure 1:**
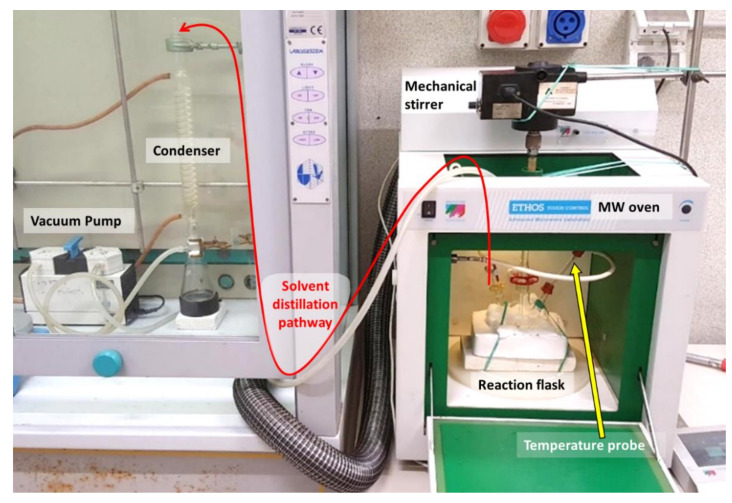
Landscape image of the microwave system modified for solvent distillation from a three-necked flask used in this study.

A Microwave apparatus (ETHOS TOUCH, Milestone s.r.l., Sorisole, Italy) was modified adequately in order to distill the solvent mixture from a three-necked flask. The experimental setup is accurately described in a previous study [17], and a landscape image of the system highlighting its main components and the pathway of solvent during distillation is presented in Figure 1. Briefly, the original microwave carousel was disassembled and replaced with a refractory material able to accommodate a three-necked flask. Agitation was performed with a mechanical stirrer that was placed above the oven: the agitation rod was inserted through the main MW hole on the top of the oven and placed in the central neck of the flask. One of the side necks of the flask was equipped with the MW temperature probe. A special homemade connection built with rubber septa and a plastic-made Pasteur pipette was used in order to keep vacuum sealing and the probe in the right position, respectively. The other side neck was equipped with a hose nozzle glass joint that was connected to the outer distillation apparatus using a rubber pipe through a second hole located on the top of the MW oven. The distillation apparatus was set up in a classic way using a vacuum pump and a condenser.

### 2.2. Materials

Ti(O-iPr)_4_ (technical grade), and an acidic mixture, denoted hereafter as AM, were provided by Colorobbia Consulting S.r.l., Sovigliana-Vinci, Italy. The composition of AM is the following: HCl (32% in water, technical grade) = 3.100%, Bi-distilled water = 96.887%, Triton X-100 (Sigma Aldrich, Darmstadt, Germany, laboratory grade) = 0.013%. The relative percent amounts of the three components have been optimized in a patent by the same company [36]. The industrial product used as a reference is commercially available by the company Colorobbia Consulting s.r.l. (Sovigliana-Vinci, Italy) with the name PH000025 and is a nanocrystalline TiO_2_ suspension, mainly in crystalline anatase form, which is used as a precursor in the preparation of products used for photocatalytic coatings on different substrates and for the functionalization of ceramic filters intended for air purification. The market of coating and/or filtration is very large since final users can be hospitals, public offices, banks, paint industries, etc.—currently, the global market of PH000025 as the principal ingredient for smart coatings is considered to reach 6 tons/y sales.

### 2.3. Procedure for TiO_2_ Nanoparticle Synthesis under Vacuum

The reaction was performed in a three-necked, round-bottomed flask placed inside the MW oven, equipped with a mechanical stirrer, a temperature probe, and a connection to the vacuum system. The latter was temporarily disconnected, and 77 mL of AM was introduced. Then, stirring was set to 600 rpm, and Ti(O-iPr)_4_ (25 mL) was added dropwise over 5 min. Finally, the connection to the vacuum was attached, the necks fixed with plastic joints, and the MW oven was closed. The vacuum pump was switched on, and the reaction mixture was then irradiated with MW at a maximum power of 500 W under vacuum, in the experimental conditions detailed in Table 1, where the product’s appearance at the end of the reaction (as powder or suspension) is reported as well.

Materials obtained as powders were divided into two portions: half of the material was left as a powder, and the other half was resuspended in bi-distilled water in order to obtain a suspension having 6 wt% concentration of TiO_2_ in water, which is the same concentration of the corresponding commercially available product cited before. This operation was made by adding the powder to water under stirring and stirring the mixture for 1–1.5 h.

Materials obtained as gel or suspensions were just diluted with bi-distilled water in order to reach the desired 6 wt% concentration of TiO_2_ in water.

All the suspensions were aged for 24 h before performing the first dynamic light scattering (DLS) measurement with the procedure indicated in Section 2.4.1. The appearance of all suspensions was cloudy, but homogeneous, without any precipitate at the bottom.

All the suspensions showed pH = 1.

### 2.4. Analytical Techniques Used

#### 2.4.1. Size Distribution

Nanoparticle size distribution of the obtained suspensions was evaluated with dynamic light scattering (DLS), using a Zetasizer S Nano ZS instrument (Malvern Panalytical Ltd., Malvern, UK). Since a high dilution is required for this analysis, the obtained 6 wt% TiO_2_ suspensions were shaken to ensure homogeneity, then a sample of the same (0.5 mL) was taken off and further diluted 1/100 (vol/vol) with bi-distilled water in a volumetric flask just before the measurements. These were carried out in a Malvern disposable polystyrene cuvette (DTS0012) on 1 mL of a diluted sample. Each measure was performed using the following instrumental parameters:

Material: Polystyrene latex;

Dispersant: Water;

Temperature: 25 °C;

Measurement angle: 173° backscatter;

Number of measurements: 5;

Number of runs per measurement: 10 runs of 10 s each.

Results, expressed in Z-average hydrodynamic diameter, are reported as the mean of the five measurements performed. The corresponding errors reported are expressed as standard deviations.

#### 2.4.2. XRD

The obtained 6 wt% TiO_2_ suspensions were dried at 50 °C for three days and then at 100 °C for six hours before analysis. The crystalline phase composition of the samples after drying was determined by X-ray powder diffraction (XRPD) using an X-ray diffractometer (model X’PERT PRO Philips, Amsterdam, NED) operating in the Bragg Brentano reflection mode equipped with a Ni-filtered Cu–Kα radiation. Each measure was performed in the 3° ≤ θ ≤ 140° angular range, with a step size of 0.00167113°, a scan speed of 0.104445°/s, and a time per step of 20.32 s.

Pattern analyses were performed using X’pert Highscore plus software, and the ICSD database was used to retrieve reference patterns of the three TiO_2_ crystalline phases anatase, brookite, and rutile. Rietveld refinement method was used to quantify the relative amounts of the different TiO_2_ polymorphs and their crystal parameters such as crystallite size. Rietveld analysis was performed using the reference JCPDS cards of, respectively, anatase (ICDD PDF No:21-1272), brookite (ICDD PDF No:29-1360), and rutile (ICDD PDF No:21-1276). The assessment of the quality of a Rietveld fit was performed by viewing the observed and calculated patterns graphically, ensuring that the model is chemically plausible. Generally, the error on the polymorph distributions and on particle size measurement is below 1% using this technique. XRD pattern of reference sample PH000025 is given in Figure S1 of Supplementary Materials.

#### 2.4.3. TEM Observations

Transmission electron micrographs (TEM) were obtained using a Transmission electron microscope (Talos™ F200S G2, Thermo Fisher Scientific, Waltham, MA, USA) operating at 200 kV, equipped with an integrated system for X-ray energy-dispersive spectroscopy (EDS). The system uses two silicon drift detectors (SDD) for enhanced sensitivity and elemental mapping capabilities (up to 105 spectra/sec). Samples were prepared by evaporating the dilute suspensions in water onto Formvar^®^ coated Cu grids. Carbon coating was adopted to enhance micrographs resolution. The stability of the specimen’s material under the scanning electron beam was checked by comparing the surface morphology before and after the focusing process. The d spacings were obtained from the selected area electron diffraction (SAED) pattern after calibration on the Au pattern. The *d_hkl_* distances were calculated as average distance over five consecutive *hkl* planes, using a CETA 16M digital camera, after extracting an area of interest from the HR–TEM images with fast FT analysis.

#### 2.4.4. BET Surface Area

Specific surface area (S_BET_) was measured by the Brunauer–Emmett–Teller (BET) method using an adsorption analyzer (ASAP 2020, Micromeritics Instrument Corporation, Norcross, GA, USA), using N_2_ as the adsorbate gas. The samples were degassed at 100 °C for 10 h before the analyses, and then, the nitrogen adsorption isotherms were obtained at 77 K for each sample. Parameters useful for surface area determination were adjusted assuming non-porous and spherical particles, as observed with HR–TEM.

### 2.5. Photocatalytic Tests

Samples’ photocatalytic activities were evaluated by depletion of nitric oxide under UV-A irradiation. The apparatus and the test execution used for the analyses are patented by Ce.Ri.Col. Colorobbia Research Center, Colorobbia Consulting S.R.L., Sovigliana-Vinci, Italy, and are described in detail elsewhere [17,37]. Briefly, the apparatus consists of a closed reaction chamber where moisture and NO are introduced, and their concentrations are controlled automatically through a given software program. This chamber is equipped with a UV lamp (Osram Ultravitalux 300 W, Osram S.p.A., Milano, Italy), used as a UV-A source that has an irradiance of 50 W/m^2^ at a distance of 12 cm. In each experiment, 1.15–1.20 g of titania suspension were applied on a 10 × 10 × 0.8 cm marble slab and dried in an oven at 50 °C. Then, the photocatalytic test was started with an initial concentration of nitric oxide of 500 ± 50 ppbv. All tests were performed at room temperature, and the relative humidity was kept around 50%. The photocatalytic efficiency of the materials was evaluated using a chemiluminescence NO–NO_2_–NO_x_ analyzer (Model 42i, Thermo Fisher Scientific, Waltham, MA, USA), performing a measurement every 15 min in order to follow the reaction kinetics. Results were compared with the ones obtained using an untreated marble substrate as a reference. The results of NO depletion values are expressed as a percentage and not as concentrations, in order to obtain values independent from the initial NO concentration [38].

## 3. Results and Discussion

### 3.1. DLS Results for Long-Term Aging

In Table 2, the DLS results after 24 h of aging on the average nanoparticle size of the five batches produced are provided and compared with the corresponding industrial product (entry **1**). The results clearly indicate that the two reactions carried out at 60 °C provide the best results in terms of lower nanoparticle size (entries 3 and 4), even better with respect to the industrial product. Additionally, one of the reactions carried out at 70 °C yields a result comparable with the industrial product (compare entry 5 and 1), while the other reaction carried out at 70 °C (entry 6) and the reaction carried out at 50 °C (entry 2) yielded poorer results. Comparing these results with the final appearance of the reaction mixture (as detailed in Table 1), we can infer that reactions providing large nanoparticle size appeared as thick white suspensions, while the others appeared as cloudy. In the case of the reaction carried out at 50 °C, probably this temperature is not high enough to produce nanoparticles of a small size. Considering the trend obtained in entries 3–5, the result of entry 6 instead is much less explicable. It is possible that despite the reaction temperature being only 10 °C higher than the one of entry 4, drying the material completely at this temperature resulted in crystal growth or product agglomeration. If we compare the results of entries 3 and 5, we can observe that there is a considerable difference in results by increasing reaction temperature by only 10 °C, leaving untouched all the other reaction conditions. This means that such a small difference in temperature might have a large influence on the reaction outcome for this process. Nevertheless, the difference between entries 4 and 6 is much higher, and the reason for this is still unclear.

The result obtained for TiO_2__60_Powd is particularly valuable especially if considered that the product was obtained in the form of a powder. In fact, drying a nanoparticle suspension often results in an increase in nanoparticle size, due to agglomeration that occurs during solution concentration [39]. Remarkably, instead, if this powder is added to water, it apparently dissolves and suddenly results in a 21.67 nm Z-average value (not reported in the table). After one day of aging, the value of 17.29 nm was reported, shown in Table 2. This means that, for some reason, agglomeration upon concentration apparently does not occur; it occurs instead in the case of entry 6. The difference between entries 3 and 4 is quite low and can be due just to serendipity, so we can consider that the powder, and the suspension obtained from this, preserve the very small size obtained during the synthesis at 60 °C. The “real” size obtained at 70 °C, instead, can be assumed the one shown by entry 5, and it is double with respect to what is obtained at 60 °C, while in entry 6, aggregates are probably formed.

**Figure 2:**
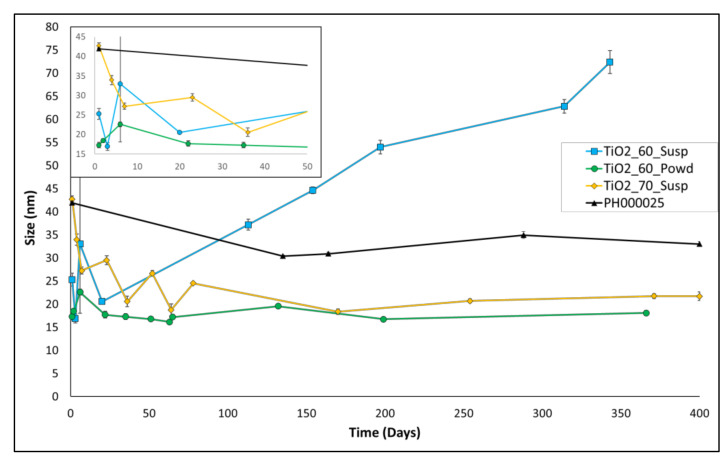
Long-term aging of entries 3–5 of Table 2, compared with the one of the industrial product PH000025 (entry 1 of Table 2). The detail of the first 50 days of aging is given in the inset.

The size over time of the three best entries of Table 2 (entries 3–5) was then monitored, in order to check suspension stability as aging proceeds, and compared with the size of the standard product PH000025. The suspensions obtained after the syntheses were, therefore, aged for approximately 350–400 days, and the results are presented in Figure 2.

Here, the stability of all three suspensions is good but with different trends. Particularly, TiO_2__60_susp shows an initial small size value; however, the average size of the nanoparticles in this batch starts increasing slowly but continuously by the 20th aging day onward. TiO_2__70_susp, conversely, shows the largest size at the beginning of the aging, displaying then a slow but constant decrease in size (despite some oscillations) and reaching a stable size of 20 nm from the 170th day onward. The most stable suspension is TiO_2__60_Powd, with an average size value of 21.67 nm at the beginning and 19.57 nm at the end of the considered aging time.

This remarkable result allows us to state that this entry can be considered the best among all of the prepared suspensions. In fact, in this preparation, we were able to directly obtain, in a short time and with a simple procedure, TiO_2_ nanopowders with a small size that can be easily resuspended in water, to yield stable suspensions without any size enlargement over time. This procedure can, therefore, be useful both for the direct synthesis of powders and for the preparation of suspensions, if needed. The reduced time of production if compared with conventional methods (Figure S2 in Supplementary Materials for further details) provides great advantages for an eventual industrial application: in this way, a reduction in costs and less environmental impact of the entire process are possible. Moreover, the possibility to produce a powder that yields a stable suspension upon reconstitution eases stocking and transport operations, because this opens the possibility to create aqueous suspensions directly on site, granting a product that preserves the same features.

### 3.2. DLS Results for Reaction Reproducibility

**Figure 3:**
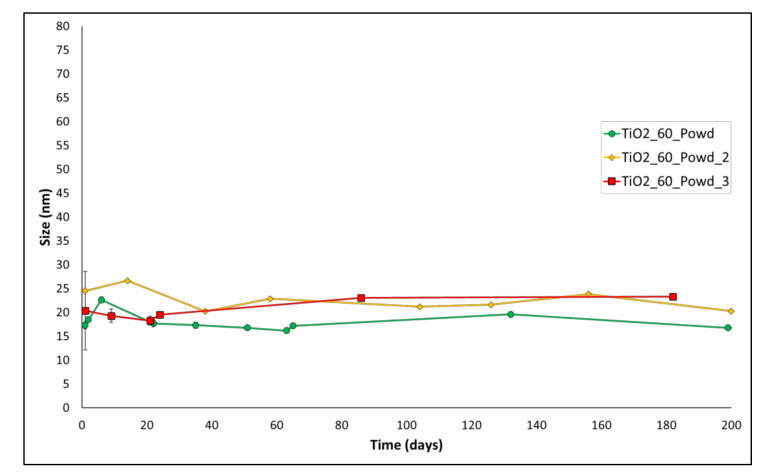
Aging of entries 1–3 of Table 3.

In order to verify the robustness of this procedure, we repeated the reaction several times to be able to verify its reproducibility. Results are presented in Table 3 and Figure 3. Table 3 shows the results of Z-average size after 24 h of aging of the suspensions. Remarkably, the reaction shows good reproducibility, with all the size values that lay between 17.29 nm and 24.50 nm. Long-term aging of these batches (Figure 3) demonstrates good stability for all the batches up to 180 days, with only small oscillations and differences between different batches. This confirms the robustness of the developed process.

### 3.3. Products Features

#### 3.3.1. XRD Analysis

**Figure 4:**
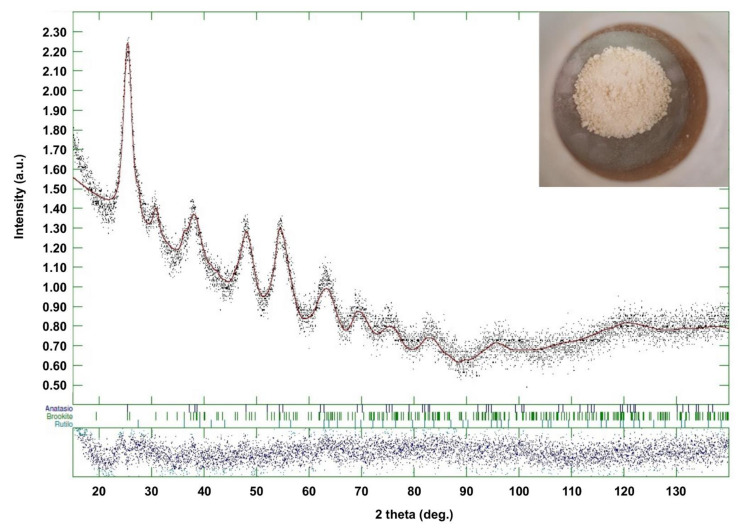
XRD spectrum of TiO_2__60_Powd batch. The inset shows a photograph of the batch.

Figure 4 shows the XRD trace for the sample TiO_2__60_Powd, compared with patterns retrieved for anatase, brookite, and rutile. Broad peaks obtained are due to the nanocrystalline nature of the material. It is, however, possible to identify anatase as the main phase, and some brookite peaks are also visible (e.g., the peak at 32°). Additionally, Rietveld refinement identifies the rutile phase as a component of this mixture, despite in low amounts, for all samples (Table 4). This mineralogic composition is in accordance with other results reported for the sol–gel synthesis of TiO_2_ nanoparticles in acidic conditions [40,41,42]. The percent phase abundances of the three TiO_2_ polymorphs and their crystal sizes are provided in Table 4 for each of the three repetitions of the best synthesis and are in accordance with the mineralogic composition of the reference material PH000025, whose XRD pattern is reported in Figure S1 of Supplementary Materials. Only the sample TiO_2__60_Powd shows a slightly higher content in anatase and lower in brookite, with brookite crystallite size being larger with respect to the reference one. All other data obtained lay within the experimental error of the technique.

#### 3.3.2. TEM Analysis

**Figure 5:**
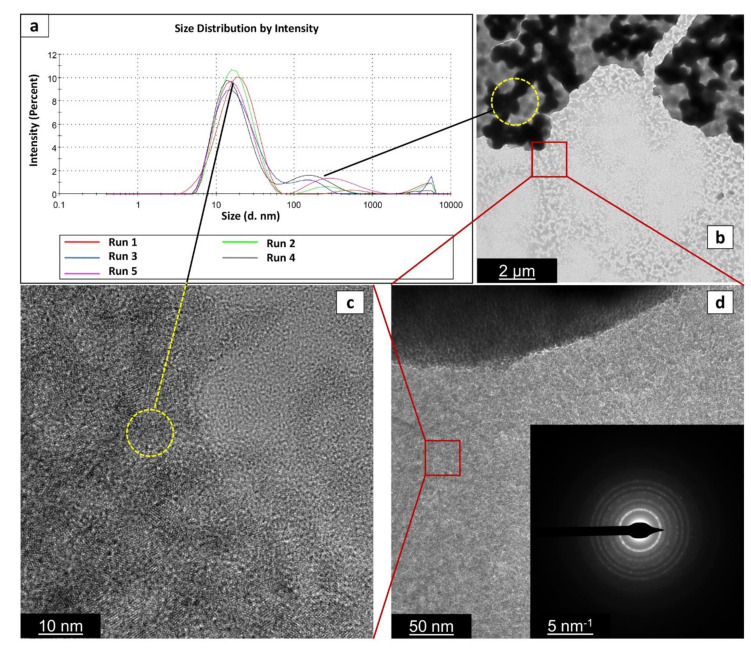
Particle size distribution via DLS analysis (**a**), TEM (**b**), and HRTEM (**c**,**d**) images of the sample TiO_2__60_Powd. With black lines and yellow circles, we indicate that images in (**b**,**c**) can provide a visual representation of the two peaks observed in (**a**–**c**), and (**d**) images were taken in the same area of the sample progressively magnified (indicated by red squares). Inset in (**d**) gives the selected area electron diffraction (SAED) image of the red squared area in (**d**).

**Figure 6:**
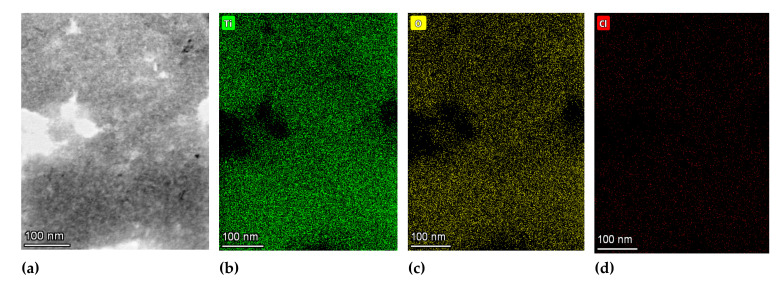
TEM bright-field image (**a**) and elemental distribution maps for Ti (**b**), O (**c**), and Cl (**d**) on an area of sample TiO_2__60_Powd. Denser fractions of the sample appear as more intense colors.

Figure 5 shows the TEM micrograph of TiO_2__60_Powd nanoparticles at different magnifications and the correlation with the particle size as collected by the DLS grain sizer by repeated preparations. TEM observation, run on dried samples, exhibit a slightly different particle size distribution with respect to what is observed in DLS measurements taken on the suspended materials, due to the high-vacuum conditions required for TEM samples preparation. Probably, most of the observed agglomeration in TEM images is only due to this fact. The particles are observed in Figure 5 to be aggregated in grains with 500–600 nm in diameter: these are, nevertheless, formed by well-dispersed spherical shape nanoparticles with 5–10 nm in size, clearly distinguishable on the aggregate’s surface. The primary particles are found to be single crystals with particle sizes of less than 10 nm, as visible in Figure 5c, and similarly to the commercial product. The SAED (inset in Figure 5d) confirms the high crystallinity of the finer particles to be a mixture of TiO_2_ anatase and brookite phases. The measured lattice fringe of 0.230 nm corresponds to the d spacing of the (004) planes of anatase [43,44], while in the SAED among the anatase diffraction rings (2.56; 3.10; 4.40 nm^−1^), we detected the brookite one at 3.50 nm^−1^ [45]. The chemical composition and element distribution in sample TiO_2__60_Powd are reported in Figure 6. Only three elements are found: Ti, O, and Cl, and their distribution appears to be homogeneous in the sample, with Cl being present at a level of a contaminant (2.73 ± 0.66%) in the TiO_2__60_Powd sample, as shown in the EDS map of Figure 6d, confirming that HCl remains as an impurity associated with this preparation method.

### 3.4. Results of Photocatalytic Tests (NO Removal) and BET Analysis

**Figure 7:**
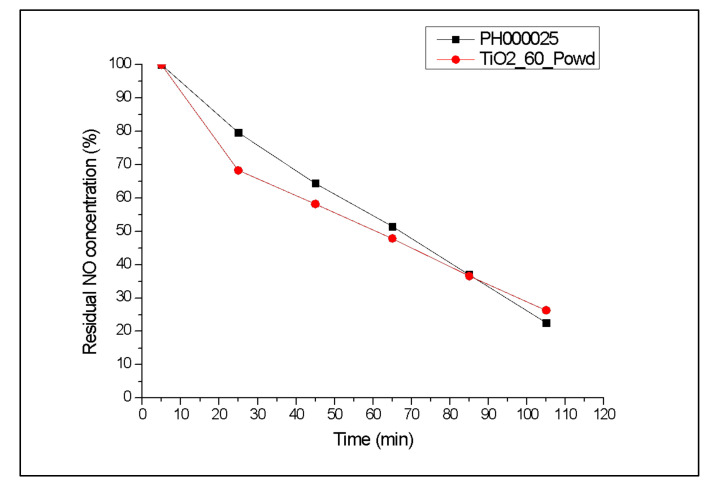
Percentage of residual NO concentration provided by sample TiO_2__60_Powd, compared with a commercially available industrial product.

Once the possibility to reproduce suspension’s stability was confirmed, and mineralogic composition and morphology of the industrial product with the simple and fast MW synthesis were reported, the photocatalytic activity of the sample TiO_2__60_Powd was examined in order to compare NO depletion efficiency of the sample with the one shown by the industrial product PH00025. Tests were carried out as reported in Section 2.5 [17,36]. Kinetic trends of residual NO concentration obtained during photocatalytic tests on samples PH000025 and TiO_2__60_Powd are reported in Figure 7, and the percentage of NO depletion after 105 min is also reported in Table 5. The photocatalytic efficiency of TiO_2__60_Powd after 105 min is comparable with the one shown by the industrial product. Moreover, TiO_2__60_Powd shows a faster kinetic in the initial 25 min; then, the NO degradation rate slightly decreases, compared with the industrial product, to ultimately yield a slightly lower result with respect to PH000025. Above all, at the end of the considered time, the photocatalytic activity of the synthesized sample is comparable to the one shown by the industrial product, and this occurs even though the BET surface area of TiO_2__60_Powd is slightly lower than the one of PH000025 (Table 5) [40,46,47].

As mentioned in the Materials and Methods Section, the final pH of all suspensions is around 1, even for the ones obtained from the powders that were synthesized and dried under vacuum. This fact, as well as considering the detection of chlorine in TEM elemental maps, suggests that TiO_2_ nanoparticles are protonated during synthesis; thus, HCl is not just a reaction catalyst or a peptization agent but also remains trapped in the synthesized material, and it is not removed as a gas under vacuum as expected. The influence of this HCl presence on photocatalysis is still unclear in the literature: There are only a low number of papers discussing its effect on photocatalysis, and some of them report it is detrimental [48,49], while others report it is beneficial [50,51,52,53]. HCl might be partly responsible for the low nanoparticle size [54,55,56], and there might be a correlation between HCl and surface properties that deeply influence photocatalysis [57,58,59,60,61,62,63]. A deep understanding of the role of the trapped HCl on the photocatalytic properties of this material is out of the scope of this work, but this phenomenon might deserve more attention in the future since, in our case, it does not seem to be detrimental for photocatalysis.

## 4. Conclusions

In summary, we disclosed a simple, fast, and efficient synthesis of TiO_2_ nanopowders carried out under vacuum in a one-pot, one-step approach with the aid of microwave irradiation. Combining the benefit of a volumetric and fast heating profile under microwave irradiation for the synthetic step, we managed to create crystalline TiO_2_ domains at a nanometric scale. Concurrently, MW heating and vacuum contributed to fast solvent evaporation that directly furnished the nanopowders in 40 min, starting from cheap and commercially available precursors. The powders obtained in this way proved to be easily resuspended in water to yield stable suspensions over time (no significant changes in size were observed up to 365 days of aging). The reaction is reproducible, and all of the batches produced with this procedure showed the same stability. The XRD of the material revealed the nanopowder to be a mixture of the three main TiO_2_ crystal phases: anatase, brookite, and rutile, with anatase being the most abundant. TEM analyses revealed very small crystallites and electron diffraction confirmed the primary crystals to be in the range 5–10 nm. Elemental analysis carried out with TEM revealed chlorine atoms uniformly distributed in the powders, proving that HCl, used for peptization during synthesis, has been trapped into the material. A possible protonation of nanoparticles was then speculated, and this also justifies the acidic pH recorded by the suspensions obtained from the powders. Despite a slightly lower BET surface area, the material synthesized with this procedure showed comparable photocatalytic performance with respect to a commercially available product; therefore, this can be considered an example of process intensification and greener synthesis for the given product. We have proved that nanopowder’s suspension quality is maintained during the fast (max 40 min) one-pot, one-step synthesis. The disclosed procedure, therefore, offers an easy way to produce both powders and suspensions of photocatalytically active TiO_2_ nanoparticles that can be used for many applications. The advantages of the present procedure become evident in view of a possible process scale-up, in terms of its time-saving process, easy operations, and safety, contributing to a leaner production and supply chain for this important photocatalytic material.

## Figures and Tables

**Table 1:** Reaction conditions and product appearance of the materials synthesized in this study.

Name	Temperature	Time	Final Appearance	Suspension Appearance
**TiO_2__50_Susp**	50 °C	30 min	White suspension	White suspension
**TiO_2__60_Susp**	60 °C	25 min	Thick white gel	Cloudy suspension
**TiO_2__60_Powd**	60 °C	40 min	Beige powder	Cloudy yellow suspension
**TiO_2__70_Susp**	70 °C	25 min	Thick white gel	Cloudy suspension
**TiO_2__70_Powd**	70 °C	40 min	Beige powder	White suspension

**Table 2:** DLS measurements on the 5 batches described in this paper after 24 h aging. Errors reported in the table are expressed as standard deviation.

Entry	Name	Z-Average (24 h of Aging)
**1**	PH000025 (Industrial product)	41.9 ± 0.3 nm
**2**	TiO_2__50_Susp	273 ± 68 nm
**3**	TiO_2__60_Susp	25 ± 1 nm
**4**	TiO_2__60_Powd	17.3 ± 0.4 nm
**5**	TiO_2__70_Susp	42 ± 1 nm
**6**	TiO_2__70_Powd	215 ± 13 nm

**Table 3:** DLS measurements for the reproducibility of TiO_2__60_Powd after 24 h of aging. Errors reported in the table are expressed as standard deviation.

Entry	Name	Z-Average (24 h of Aging)
**1**	TiO_2__60_Powd	17.3 ± 0.4 nm
**2**	TiO_2__60_Powd_2	24 ± 2 nm
**3**	TiO_2__60_Powd_3	20 ± 1 nm

**Table 4:** Mineralogic composition of the samples TiO_2__60_Powd.

Batch	Anatase (%) (Crystal Size (nm))	Brookite (%) (Crystal Size (nm))	Rutile (%) (Crystal Size (nm))
PH000025 (Industrial Product)	63 (5)	32 (4)	5 (9)
TiO_2__60_Powd	71 (5)	24 (31)	4 (6)
TiO_2__60_Powd_2	64 (2)	33 (4)	3 (22)
TiO_2__60_Powd_3	62 (4)	30 (3)	8 (7)

**Table 5:** BET surface area of sample TiO_2__60_Powd, compared with the commercially available industrial product PH000025, and percentage of NO depletion provided by sample TiO_2__60_Powd, compared with the same.

Batch	NO Depletion after 105 min	BET Surface Area
PH000025 (Industrial product)	78%	192.7869 m^2^/g
TiO_2__60_Powd	75%	123.2593 m^2^/g

## Data Availability

The data presented in this study are available on request from the corresponding author.

## Supplementary Materials

The following supporting information can be downloaded at: https://www.mdpi.com/article/10.3390/nano12010149/s1, Figure S1. XRD pattern of reference material PH000025. Figure S2. Example process schemes of (a) conventional TiO_2_ synthesis and (b) the MW-assisted vacuum synthesis of the same material developed in this work. The main advantages in time saving are in the fastest MW heating and in the reaction step, which is performed at the same time of distillation, thus reducing reaction times in the second process.
