# Supplementary material for: Microwave-Assisted Vacuum Synthesis of TiO2 Nanocrystalline Powders in One-Pot, One-Step Procedure
Source: Nanomaterials (Basel). 2021 Dec 31;12(1):149. doi: 10.3390/nano12010149 (PMC8746694; doi:10.3390/nano12010149)
Supplement: Supplementary file 1 [file nanomaterials-12-00149-s001.zip › nanomaterials-1491523-supplementary.pdf]

nanomaterials

*Supplementary Materials*

# Microwave-Assisted Vacuum Synthesis of $TiO_2$ Nanocrystalline Powders in One-Pot, One-Step Procedure

**Enrico Paradisi [1,*], Roberto Rosa [2], Giovanni Baldi [3], Valentina Dami [3], Andrea Cioni [3], Giada Lorenzi [3] and Cristina Leonelli [1]**

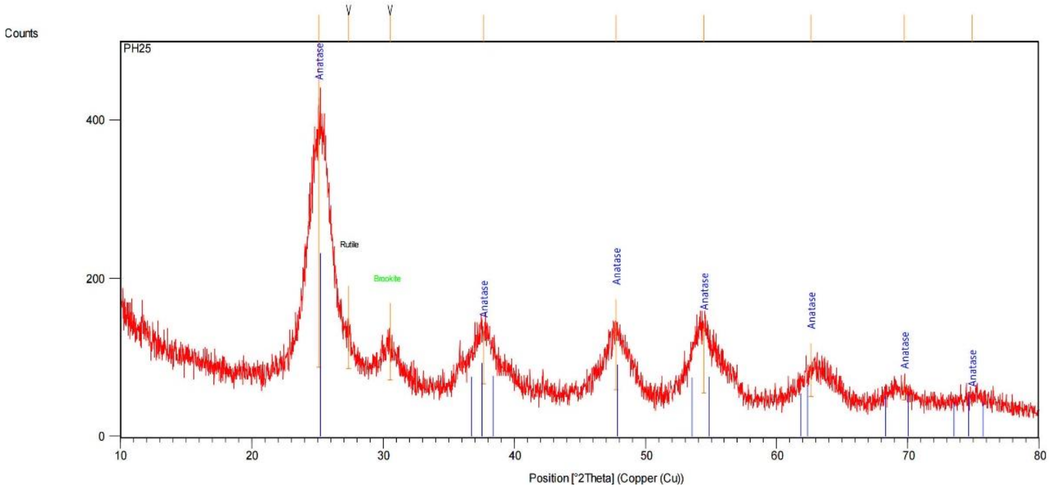


**Figure S1.** XRD pattern of reference material PH000025.

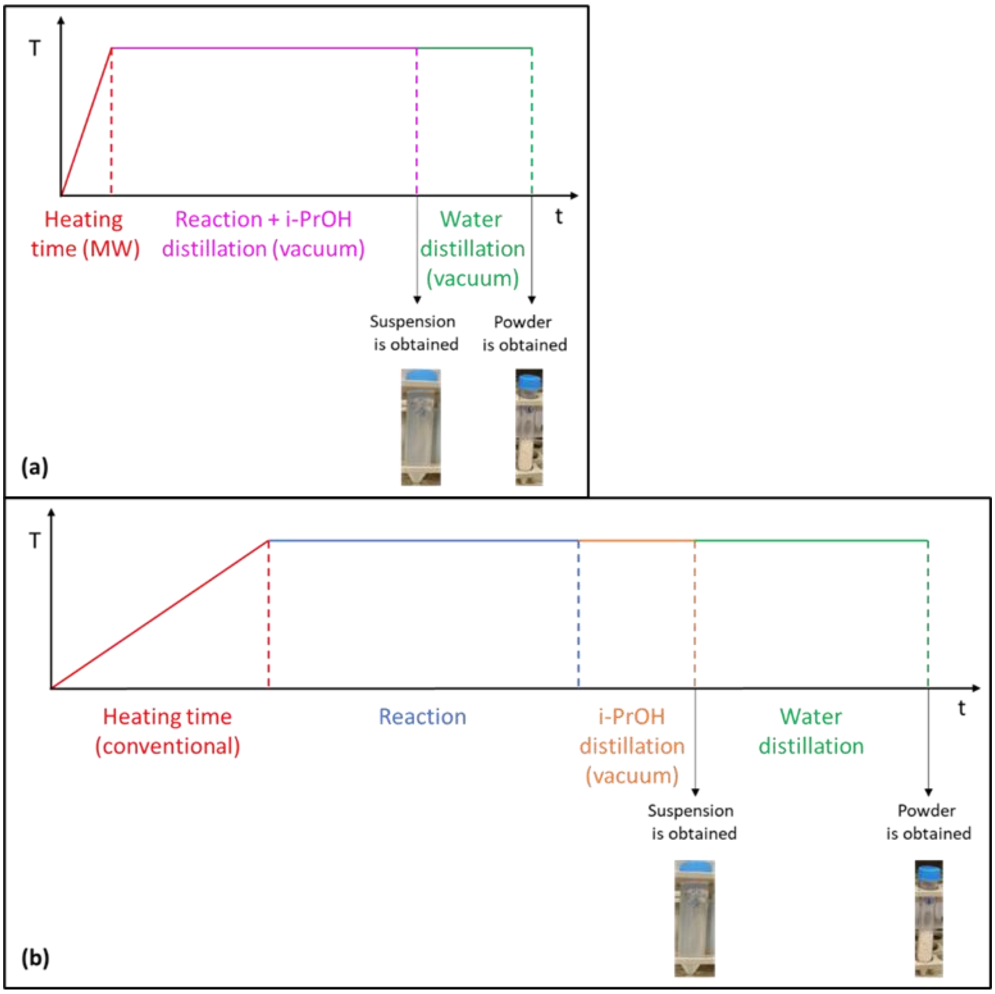


**Figure S2.** Example process schemes of (**a**) conventional $TiO_2$ synthesis and (**b**) the MW-assisted vacuum synthesis of the same material developed in this work. The main advantages in time saving are in the fastest MW heating and in the reaction step, which is performed at the same time of distillation, thus reducing reaction times in the second process.
